# Konjac Glucomannan–Montmorillonite Hybrids as a Gut-Targeted Therapy for Addressing Diet-Induced Obesity in Mice

**DOI:** 10.3390/nu18081298

**Published:** 2026-04-20

**Authors:** Amin Ariaee, Hannah R. Wardill, Alex Hunter, Anthony Wignall, Aurelia S. Elz, Amanda J. Page, Clive Prestidge, Paul Joyce

**Affiliations:** 1 School of Pharmacy and Biomedical Science, Adelaide University, Adelaide 5000, Australia; amin.ariaee@adelaide.edu.au (A.A.); hannah.wardill@adelaide.edu.au (H.R.W.); alex.hunter@adelaide.edu.au (A.H.); anthony.wignall@adelaide.edu.au (A.W.); aurelia.elz@sa.gov.au (A.S.E.); amanda.page@adelaide.edu.au (A.J.P.); clive.prestidge@adelaide.edu.au (C.P.); 2 Supportive Oncology Research Group, Precision Cancer Medicine, The South Australian Health and Medical Research Institute, Adelaide 5000, Australia

**Keywords:** obesity, konjac glucomannan, montmorillonite, gut microbiota, metabolic dysfunction, inflammation

## Abstract

**Background/Objectives**: The growing prevalence of obesity necessitates innovative gut-targeted material strategies to modulate diet-associated metabolic dysfunction. This study investigates a spray-dried konjac glucomannan–montmorillonite (KGM-MMT) hybrid designed to integrate fermentable polysaccharide properties with luminal lipid-adsorptive clay functions within a single micro-engineered formulation. **Methods:** In HFD-fed mice treated for 42 days with 2% *w*/*w* KGM-MMT, cumulative body weight gain was attenuated by 7.6%, with an AUC of 5094 ± 52.95, compared to 5513 ± 81.35 in HFD controls (*p* < 0.0001). **Results:** Serum IL-6 concentrations were reduced by 97% (*p* = 0.0002), while blood glucose decreased by 46% (*p* < 0.0001); these effects were greater than those observed with MMT (24%, *p* = 0.0271) and KGM (16%, ns). Gut microbiota profiling demonstrated a significant 6.2-log_2_-fold increase in *Lactobacillaceae* (*p* = 0.023) and a 2.4-log_2_-fold increase in *Enterococcaceae* (*p* = 0.015) following KGM-MMT treatment. Functional shifts inferred from 16S rRNA gene-based prediction indicated a 1.9-fold increase in short-chain fatty acid-related pathways and a 5.4-fold increase in bile acid deconjugation pathways. **Conclusions:** Although the KGM-MMT hybrid did not consistently outperform its individual components across all endpoints, it consolidated complementary KGM- and MMT-associated effects within a single dosage form. These findings support spray-dried KGM-MMT as a gut-targeted biomaterial strategy that integrates multiple luminal and microbiota-associated functions within a single formulation. Future studies should define dose–response relationships, validate microbiota-derived functional predictions using higher-resolution approaches, and assess durability and safety under longer-term exposure.

## 1. Introduction

Obesity and its associated metabolic disorders continue to pose significant global health challenges, necessitating innovative gut-targeted material strategies to mitigate their impact. According to the World Health Organization, more than 1 billion people are currently living with obesity, a prevalence that has more than doubled since 1990 [1]. This epidemic carries a substantial clinical burden, including type 2 diabetes mellitus, non-alcoholic fatty liver disease, cardiovascular disease, and certain malignancies [2,3]. These conditions contribute to premature mortality and reduced quality of life. Despite available pharmacological and surgical interventions, long-term outcomes remain suboptimal for most individuals [4]. This highlights the urgent need for safe, food-grade strategies that target the underlying metabolic drivers of obesity. A growing body of evidence suggests that dietary and material-based interventions targeting the gut microbiota and systemic inflammation may hold promise in attenuating these conditions [5,6]. Konjac glucomannan (KGM), a dietary fiber derived from the tubers of *Amorphophallus konjac*, has received attention for its fermentable, prebiotic, and anti-inflammatory properties [7,8,9]. Its high viscosity and gel-forming properties contribute to delayed gastric emptying and reduced nutrient absorption, mechanisms that have been linked to attenuation of diet-induced weight gain [10]. Additionally, KGM has been demonstrated to selectively enrich the gut microbial taxa associated with improved epithelial barrier function and reduced metabolic endotoxemia [5,11].

Beyond its dietary uses, KGM has been incorporated into biomaterials, including hydrogels and wound dressings, leveraging its biocompatibility and rheological properties to support tissue regeneration and drug delivery applications [12]. However, despite its widespread use in dietary and biomedical applications, KGM’s capacity to modulate chronic low-grade inflammation, a hallmark of obesity and metabolic diseases, remains underexplored [13]. Further, the high viscosity of unmodified KGM powder lends itself to choking hazards due to its capacity to swell up to 50 times its weight in aqueous conditions, such as the stomach, posing significant dosing challenges [14]. Engineering it into hybrid, micron-sized particulate systems with complementary materials offers a strategy to improve dosing safety and gastrointestinal functionality. This approach reduces particle size and improves formulation uniformity, mitigating risks associated with conventional KGM dosing while preserving and combining its functional properties with those of complementary biomaterials.

Adsorbent clays, such as montmorillonite (MMT), provide a complementary material platform for such applications. MMT, a naturally occurring layered silicate, exhibits a high surface area and adsorption capacity, along with reported biocompatibility and the ability to influence gut luminal composition and inflammatory markers under diet-induced obesity conditions [15,16,17]. Its layered platelet architecture enables self-assembly into porous three-dimensional microstructures that maximize accessible adsorption sites [17,18]. These porous structures enhance the capacity of MMT to bind luminal compounds, and recent studies have extended this concept to the adsorption of lipolytic products within the intestinal lumen, thereby attenuating metabolic consequences associated with high-fat diets [16,17,19,20]. A previous study demonstrated that spray-dried MMT adsorbed approximately 42% of all lipid species in an in vitro lipid digestion model and attenuated diet-induced weight gain in rats to an extent comparable with orlistat, a pancreatic lipase inhibitor [17].

As such, the current study investigates whether micro-structured KGM-MMT hybrids can integrate complementary gut-active mechanisms within a single particulate formulation. This approach employs spray drying to combine KGM’s fermentable and viscosity-driven functions with MMT’s luminal adsorption capacity within a single hybrid material. By leveraging MMT’s porous microstructures and KGM’s fermentable polysaccharide profile, the hybrid formulation is designed to restrict luminal lipid digestion in the small intestine while supporting microbiota-associated metabolic functions in the gut. To our knowledge, this is the first study to examine spray-dried KGM-MMT hybrids as a food-grade biomaterial platform that spatially integrates lipid-restrictive and microbiota-associated functions within a diet-induced obesity model.

## 2. Materials and Methods

### 2.1. Materials

KGM was purchased from Sigma-Aldrich (Castle-Hill, Australia). MMT was supplied by Pharmako Biotechnologies (Frenchs Forrest, Australia). Medium-chain triglycerides (MCT; Miglyol^®^ 812), consisting of caprylic and capric acids in a 50:50 (*w*/*w*) ratio, were purchased from Hamilton Laboratories (Adelaide, Australia). Male BALB/c mice (6–8 weeks old at study initiation) were acquired from the breeding program at the South Australian Health and Medical Research Institute Centre (Adelaide, Australia). Male mice were selected to reduce variability associated with estrous cycling and sex-dependent differences in bile acid metabolism and adiposity [21,22]. All chemicals and solvents used in this study were of analytical grade and ultra-pure water (Milli-Q, Burlington, MA, USA) was utilized throughout the experimental procedures.

### 2.2. Fabrication of KGM-MMT Hybrids via Spray Drying

This section describes the fabrication of hybrid microparticles by co-spray drying KGM and MMT. The aim was to combine the complementary functions of both materials in a single particle. Spray drying is a scalable top-down processing method commonly employed for polysaccharide–inorganic composite biomaterials [23,24]. Briefly, an aqueous dispersion of KGM at 2% *w/v* was formed by dispersing 20 g of KGM in 1 L of Milli-Q water. The solution was stirred at room temperature for 30 min. Simultaneously, 20 g of MMT was dispersed in 1 L of Milli-Q water under similar conditions. The KGM and MMT dispersions were combined at a 1:1 mass ratio to promote homogenous hybrid formation during atomization. The resulting mixture was spray-dried using a mini Spray-Dryer B-290 (Büchi, Flawil, Switzerland) under the following conditions: inlet temperature of 200 °C, outlet temperature of 105 °C, aspirator setting at 100%, nozzle cleaning setting at 9, compressed air flow rate of 40 mm, and product flow rate of 7 mL/min. Spray drying parameters were selected to promote rapid solvent evaporation and formation of micron-scale, porous hybrid particles [25].

### 2.3. Morphological Analysis via Scanning Electron Microscopy (SEM)

Scanning electron microscopy (SEM) was used to compare the surface morphology of KGM, MMT, and the spray-dried hybrid. It provided qualitative evidence of hybrid formation and particle structure relevant to gastrointestinal function. The surface morphology and qualitative size characteristics of KGM, MMT, and spray-dried KGM-MMT hybrids were examined using a Zeiss Crossbeam 540 scanning electron microscope (Oberkochen, Germany). For SEM sample preparation, a small quantity of each sample was adhered to carbon tape mounted on SEM stubs. The samples were then coated with a platinum layer approximately 10–20 nm thick using a sputter coater. Imaging was performed at an accelerating voltage of 1–2 kV. SEM micrographs were processed and analyzed with AztecOne 4.0 software (Oxford Instruments, Abingdon, UK).

### 2.4. Particle Size Analysis

Particle size analysis quantified the size reduction from spray drying and confirmed that the hybrid particles are in the micron range for gastrointestinal delivery. The particle size of KGM, MMT, and spray-dried KGM-MMT hybrids were determined using laser diffraction (Mastersizer, Malvern Instruments, Worcestershire, UK). Samples were dispersed in an aqueous buffer, simulating small intestinal pH conditions (pH 6.5), under continuous stirring for 60 min. The particle size distribution was characterized by the D_50_ value, which represents the median particle diameter where 50% of the particles by volume are smaller than this size. Measurements were performed in triplicate.

### 2.5. In Vitro Simulated Gastrointestinal Lipolysis Assay

An in vitro intestinal lipolysis model was used to evaluate the effect of KGM, MMT, and spray-dried KGM-MMT hybrids on lipid digestion under simulated fasted and fed state simulated intestinal conditions (pH 6.5) [17,18]. Briefly, the simulated fasted and fed state gastric (pH 1.6) and intestinal media (pH 6.5) were prepared using a 50 mM Tris-maleate buffer. Media were freshly prepared and used within 48 h. Pancreatin extract was prepared by dissolving 2 g of pancreatin powder in 10 mL of the respective media (fasted state simulated intestinal fluid (FaSSIF) or fed-SSIF (FeSSIF), pH 6.5) and centrifuging at 2268× *g* for 20 min at 4 °C. The supernatant was collected and stored at 4 °C until use. The digesting lipid, MCT (625 mg), was dispersed in 20 mL of FaSSIF or FeSSIF by continuous stirring at 600 rpm for 10 min in a thermostated glass reaction vessel maintained at 37 °C. KGM, MMT, or spray-dried KGM-MMT hybrids were incorporated into the dispersion at 10% *w*/*w* relative to the lipid content. The pH of the lipolysis media was adjusted to 6.5 ± 0.01 using 0.1 M sodium hydroxide or hydrochloric acid. Lipolysis was initiated by adding 2 mL of pancreatin extract (equivalent to 2000 tributyrin units) to the reaction vessel. The liberation of free fatty acids (FFAs) was monitored for 25 min using a pH-stat titrator (902 Titrando, Metrohm, Herisau, Switzerland), which maintained a constant pH of 6.5 by titrating 0.6 M sodium hydroxide.

### 2.6. In Vivo Study Design

A murine diet-induced obesity model was used to assess the in vivo metabolic effects of KGM, MMT, and the KGM-MMT hybrid. The primary outcome was cumulative body weight gain. Secondary outcomes included inflammation, glycemia, and body composition. The in vivo study was approved by the Animal Ethics Committee at the South Australian Health and Medical Research Institute (SAHMRI, Adelaide, Australia) (Approval Code: SAM-21-009 Approval Date: 30 March 2021) and were conducted in accordance with the NIH Principles of Laboratory Animal Care (NIH publication #85–23, revised 1985), the Australian Code for the Care and Use of Animals for Scientific Purposes (8th edition, 2013, revised 2021), and ARRIVE 2.0 guidelines [26]. Forty male BALB/c mice (6–8 weeks old at study initiation) were obtained from the SAHMRI breeding facility. Male mice were selected to reduce variability associated with estrous cycling and sex-dependent differences in adiposity and bile acid metabolism. After a one-week acclimation period, mice were randomly allocated using a computer-generated sequence into four parallel groups (*n* = 10 per group): high-fat diet (HFD; 44% kcal from fat) control, HFD supplemented with 2% *w*/*w* KGM, HFD supplemented with 2% *w*/*w* spray-dried MMT, or HFD supplemented with 2% *w*/*w* spray-dried KGM-MMT hybrids. The experimental unit for all analyses was a single animal. A chow-fed lean control was not included, as the study aim was to compare treatment-specific effects within a consistent obesogenic environment rather than normalization to a healthy baseline. Blinding was not performed during allocation, conduct of the experiment, or outcome assessment due to incorporation of treatments directly into the diets.

Mice were group-housed (three per cage) under controlled environmental conditions (12 h light/dark cycle, 22 ± 2 °C, 55 ± 10% humidity) with ad libitum access to food and water, standard bedding, and environmental enrichment. Cage positions were rotated weekly to minimize environmental bias. Animals were monitored daily for health and welfare. The intervention lasted 42 days, a duration selected based on established HFD models demonstrating metabolic and microbiota alterations within 4–6 weeks of exposure [27]. The primary outcome was cumulative body weight gain quantified by area under the curve (AUC). Secondary outcomes included serum IL-6 and IFN-γ concentrations, blood glucose, body composition, food intake, meal size, and gut microbiota composition and predicted functional pathways. During the final week, mice were individually housed in metabolic monitoring cages (Promethion, Sable Systems International, North Las Vegas, NV, USA) for continuous measurement of respiratory quotient, energy expenditure, food intake, and activity over a 24 h period for three consecutive days. Body composition was assessed non-invasively using EchoMRI (Houston, TX, USA). At study termination, mice were anesthetized with isoflurane, blood was collected by cardiac puncture for serum analyses, and animals were euthanized by cervical dislocation.

Microbiome samples failing predefined sequencing quality control thresholds were excluded prior to analysis, resulting in *n* = 8 per group for 16S-based analyses. From an initial cohort of 40 mice, 10 per group, two samples per group were excluded due to low DNA yield and failure to meet the minimum read depth of 10,000 quality-filtered reads after demultiplexing and adapter trimming. Exclusions were applied uniformly across all groups before statistical analysis and were not based on treatment outcomes. Humane endpoints were predefined as greater than 20% body weight loss or signs of severe distress; no animals met these criteria. A formal protocol was not preregistered. Data supporting the findings of this study are available from the corresponding author upon request.

### 2.7. 16S rRNA Gene Sequencing of Fecal Samples

16S rRNA gene sequencing was used to assess how treatments changed gut microbiota composition and diversity. This allowed evaluation of the formulations’ prebiotic and microbiota-modulating effects in diet-induced obesity. Following humane euthanasia, fecal samples were collected and shipped to the Australian Genomics Research Facility (Brisbane, Australia) for DNA extraction and 16S ribosomal RNA (rRNA) gene sequencing targeting the V3-V4 hypervariable regions. The resultant sequences were clustered into operational taxonomic units (OTUs) at a 97% similarity threshold using QIIME 2 and the Silva reference database (Release 138.1). OTU taxonomic assignments were determined with QIAGEN CLC Genomics Workbench Version 23.0.4 and the QMI-PTDB TaxPro index (June 2021). Microbial diversity was characterized by calculating Shannon, Chao1 indices and total OTU number for alpha diversity. Beta-diversity indices were obtained using Bray–Curtis dissimilarity, Jaccard indices, and weighted/unweighted UniFrac distances. Statistical differences in beta-diversity were evaluated via Permutational Multivariate ANOVA (PERMANOVA), and PCoA plots for each group were displayed with 95% confidence ellipses to visualize group clustering and variability. Samples failing predefined sequencing quality control thresholds were excluded, resulting in a final sample size of *n* = 8 per group. While this reduction may limit statistical power for certain microbiota endpoints, consistent directional effects across multiple analyses support biological relevance.

### 2.8. 16S rRNA Metagenomic Predictions Using PICRUSt2

Phylogenetic Investigation of Communities by Reconstruction of Unobserved States (PICRUSt) version 2.0 was used to infer the relative abundance of microbial enzymes and metabolic pathways from 16S rRNA gene profiles. Enzyme predictions were generated using the Enzyme Commission (EC) database (November 2023), and the corresponding EC numbers were subsequently applied to estimate metabolic pathway abundances via the MetaCyc Pathway Database (May 2022). It is important to note that metagenomic predictions are not a direct metagenomic or metabolomic validation.

### 2.9. Serum Pro-Inflammatory Cytokine Quantification Using ELISA

Serum cytokines were measured to assess systemic anti-inflammatory effects of each treatment. IL-6 and IFN-γ were analyzed as key pro-inflammatory markers in diet-induced metabolic dysfunction. Following centrifuging whole blood at 2000× *g* for 20 min at 4 °C, the serum fraction was carefully collected and stored at −80 °C until further analysis. Serum interleukin-6 (IL-6) and Interferon-gamma (IFN-γ) concentrations were measured using ELISA kits (ThermoFisher Scientific, Scoresby, Australia), following the manufacturer’s instructions. Quantification was carried out by generating standard curves from known IL-6 concentrations. All assays were performed in duplicates according to standardized protocols to ensure consistency and reproducibility. The limit of detection is reported as 3 pg/mL.

### 2.10. Statistical Analysis

Statistical analyses were performed using GraphPad Prism Version 10.2.0 (GraphPad Software, Boston, MA, USA), except for 16S rRNA sequencing analyses, which were conducted using QIIME2 and associated bioinformatics tools as described above. Data are presented as mean ± standard deviation (SD) unless otherwise specified. Box and violin plots display the full data range (minimum to maximum values). Normality of data distribution and residuals was assessed using the Shapiro–Wilk test. For normally distributed data, comparisons among the four dietary groups were performed using one-way analysis of variance (ANOVA) followed by Tukey’s multiple comparisons test. For non-normally distributed data, the Kruskal–Wallis test followed by Dunn’s multiple comparisons test was applied. For microbiome beta-diversity analyses, statistical differences between groups were assessed using permutational multivariate analysis of variance (PERMANOVA) based on Bray–Curtis, Jaccard, weighted UniFrac, or unweighted UniFrac distance matrices, as appropriate. Differential abundance testing at the family level was performed using Welch’s *t*-test with correction for multiple comparisons.

All statistical tests were two-tailed. Exact *p*-values are reported where possible, and statistical significance was defined as *p* ≤ 0.05. No statistical methods were used to predetermine sample size. Samples excluded following microbiome sequencing quality control (final *n* = 8 per group) were removed prior to statistical analysis and are reported transparently in the Methods. Researchers were not blinded to group allocation during outcome assessment.

## 3. Results and Discussion

### 3.1. Spray Drying of KGM with MMT Produces Microparticles

**Figure 1:**
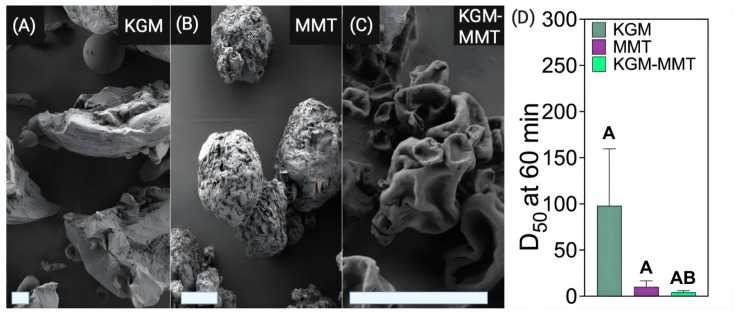
SEM images illustrate the morphological differences between KGM-MMT hybrids and their precursor materials. (**A**) KGM exhibits irregular, 20–50 µm sized porous structures. (**B**) Spray-dried MMT forms 8–10 µm sized spherical particles with a smoother surface, indicative of enhanced uniformity and dispersion. (**C**) Hybrid particles formed by spray drying KGM with MMT demonstrate KGM incorporation into the MMT matrix, resulting in 2–5 µm particles with sheet-like morphology. (**D**) Particle size analysis of spray-dried KGM-MMT dispersed in an aqueous pH 6.5 buffer reveals significantly smaller particle sizes compared to its individual precursors, highlighting the effect of spray drying on particle size reduction. Scale bars represent 10 µm. Particle size data is presented as mean ± SD (*n* = 3 per group). Statistical differences were determined using one-way ANOVA with Tukey’s comparisons post hoc. Distinct letter notation (A, B) denotes statistically different groups, where groups sharing the same letter are not significantly different.

The structural and morphological properties of KGM and MMT are central to their gastrointestinal functionality and underlie the rationale for engineering hybrid microparticles that integrate physicochemical and microbiota-mediated mechanisms. SEM analysis revealed distinct microarchitectures of KGM, MMT, and the hybrid formulation, highlighting features relevant to luminal dispersion, adsorption, and microbial accessibility. SEM imaging of KGM powder revealed amorphous particles with a porous morphology capable of forming highly hydrated networks (Figure 1A) [28,29,30]. KGM’s high water-holding capacity enhances viscosity and fermentability, facilitating microbial access and polysaccharide utilization in the colon [31,32]. These properties contribute to its role as a fermentable dietary fiber, supporting microbial metabolism in the gut. Consequently, KGM supplementation has been reported to support the expansion of commensal taxa and increase microbial short-chain fatty acid (SCFA) production [33]. However, unmodified KGM particles typically span 20–50 µm, which may limit homogeneous dispersion and microbial interaction in aqueous intestinal environments. Additionally, the pronounced swelling capacity of unmodified KGM powders has raised safety concerns related to esophageal obstruction at high doses [34]. Similarly, an in vitro fermentation study demonstrated that reducing KGM’s particle size via acid modification resulted in significantly higher substrate utilization by the inoculated fecal microbiota and enhanced SCFA production [35]. Collectively, these findings support particle-size reduction as a strategy to improve both the functional performance and safety profile of KGM-based formulations. In contrast, MMT particles are irregular and compact, characterized by a rough surface morphology that reflects the extrusion of the layered platelet structure within the aluminosilicate clay (Figure 1B).

Spray drying of KGM with MMT generated hybrid microparticles with wrinkled, sheet-like morphologies, indicative of polymer–clay integration during rapid solvent evaporation (Figure 1C). This process reduced particle size to 2 to 5 µm and improved size uniformity, consistent with polymer clay co-processing by spray drying [17,18]. It is important to note that direct evidence of stable molecular complex formation was not obtained in this study. The observed morphology therefore indicates physical hybridization rather than covalent or ionic bonding. Previous studies on biopolymer–MMT systems provide a mechanistic basis for these interactions. Polysaccharides such as carboxymethyl cellulose interact with MMT primarily through hydrogen bonding between functional groups and surface hydroxyl or interlayer water, leading to surface adsorption and partial delamination of the clay structure [36]. Similar hydrogen bonding-driven interactions have been reported in soy protein MMT and carrageenan MMT systems [37,38]. Electrostatic contributions have also been described, where positively charged domains within biopolymers facilitate association with negatively charged MMT surfaces, promoting intercalated structures [38].

Across these systems, the combined effects of hydrogen bonding, electrostatic interactions, and processing conditions such as spray drying dictate the formation of dispersed and structurally integrated composites. The current observations are consistent with physical association and co-assembly of KGM and MMT during processing. Further physicochemical characterization would be required to define the precise nature and stability of these interactions in the present system. Nonetheless, particle size analysis further corroborates these findings, as shown in Figure 1D. After 60 min of dispersion in an aqueous intestinal pH 6.5 buffer, the spray-dried KGM-MMT hybrid exhibited a median particle size of 4.19 ± 0.75 µm, corresponding to a 23-fold reduction compared to KGM (97.99 ± 30.00 µm) and a 2.5-fold reduction compared to unmodified MMT (10.29 ± 3.19 µm). It should be noted that the D50 values for KGM (97.99 ± 30.00 µm) and MMT (10.29 ± 3.19 µm) represent unprocessed raw materials dispersed under identical buffer conditions. These serve as pre-spray drying baselines for the hybrid. The KGM measurement at 60 min dispersion at pH 6.5 reflects swollen, partially hydrated particles. At this concentration and timepoint, laser diffraction detects suspended particulate material rather than a fully dissolved system [39]. This is consistent with the high molecular weight and slow dissolution kinetics of native KGM [40]. Tukey’s multiple comparisons test confirmed that the difference between KGM-MMT and KGM was statistically significant (*p* = 0.0423), while the difference between KGM-MMT and MMT was not significant (*p* = 0.9764). These reductions demonstrate that spray drying is an effective strategy for generating stable, micron-scale hybrid particles suitable for gastrointestinal delivery [17,20]. The smaller, more uniform particles increase the surface area, enhancing KGM-MMT’s gut activity. Spray-dried smectite clays have been shown to reduce in vitro fat digestion by up to 1.4-fold compared to their unmodified forms [17]. Importantly, the spray-dried hybrid material is intended to confer additive, rather than strictly synergistic, functionality by integrating lipid adsorption and fermentable substrate delivery within a single dosage form. MMT is a well-documented adsorbent for lipolytic products, such as FFAs in the small intestine, due to its high cation exchange capacity and layered structure, which predominately bind FFAs via the silanol groups at the clay’s broken platelet edges [17,18,19,20]. Spray drying optimizes these properties by improving dispersion in the gastrointestinal tract, enabling MMT to adsorb more lipolytic products and remove them from the small intestine before systemic absorption [17]. The hybridization of KGM-MMT is postulated to offer a multi-faceted effect in the gastrointestinal tract: MMT adsorbs lipolytic products in the small intestine, while KGM reaches the large intestine intact, serving as a fermentable substrate for the residing microbiota. Previous studies have demonstrated that spray drying effectively hybridizes fermentable polysaccharide-based gut-actives, such as inulin–MCT, significantly reducing weight gain in obese rats [41]. These findings establish a proof-of-concept for using spray drying to develop multifunctional biomaterials with enhanced therapeutic potential. Future studies should also assess the particle size behavior of such materials in pH buffers relevant to the gastric phase, in addition to the intestinal phase, to better understand their stability and functionality across different regions of the gastrointestinal tract.

### 3.2. KGM-MMT Hybrids Suppress In Vitro Simulated Intestinal Fat Digestion

**Figure 2:**
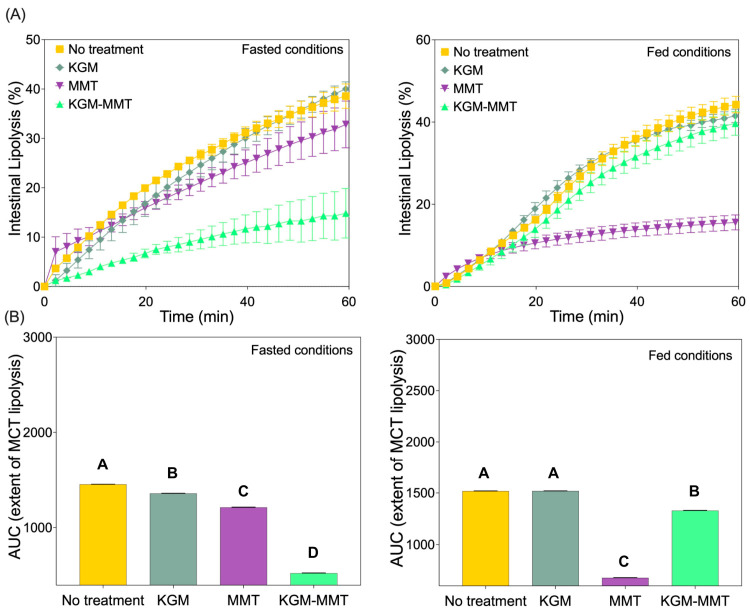
(**A**) In vitro intestinal lipolysis of MCT with KGM, MMT, and KGM-MMT hybrids under fasted (left) and fed (right) conditions. KGM-MMT hybrids show the greatest suppression of FFA release in the fasted state, while MMT is most effective in the fed state. (**B**) AUC analysis reveals reductions in lipolysis by KGM-MMT hybrids and MMT, with KGM having no effect in the fed state. AUC data is presented as mean ± SD (*n* = 3 per group). Statistical differences were determined using the Kruskal–Wallis test with Dunn’s multiple comparisons post hoc. Distinct letter notation (A, B, C, D) denotes statistically different groups, where groups sharing the same letter are not significantly different.

Under fasted conditions, the KGM-MMT hybrid significantly suppressed intestinal lipid digestion compared to KGM, MMT, and HFD controls (Figure 2A). After 60 min of in vitro MCT digestion, the free fatty acid (FFA) release for KGM-MMT was 525.1 ± 2.7 µmol, representing a 4.0-fold reduction compared to HFD controls (1451 ± 1.2 µmol, *p* < 0.0001), a 2.71-fold-greater reduction compared to KGM (1356 ± 2.1 µmol, *p* < 0.0001), and a 2.21-fold-greater reduction compared to MMT (1211 ± 2.7 µmol, *p* < 0.0001). Dunn’s multiple comparisons test confirmed significant differences between all groups, with KGM-MMT showing the most profound inhibition (*p* < 0.0001 for all comparisons). The marked reduction in FFA release observed with KGM-MMT is consistent with additive contributions from MMT-mediated lipid adsorption and KGM-induced viscosity effects that collectively limit enzyme–substrate accessibility [20,42]. Moreover, KGM particles may disrupt the lipid-in-water interface, impairing emulsification and thereby diminishing the surface area available for enzymatic action [43].

In the fed state, a different pattern was observed. MMT alone was the most effective in reducing FFA release (678.3 ± 1.5 µmol), achieving a 1.9-fold reduction compared to HFD (1518 ± 1.5 µmol, *p* < 0.0001). In comparison, the KGM-MMT hybrid reduced FFA release by 1.14-fold (1329 ± 2.3 µmol, *p* < 0.0001), while KGM alone (1519 ± 1.0 µmol) had no significant effect (*p* = 0.8720). These findings indicate that under emulsifier-rich fed conditions, MMT-driven adsorption predominates [9], whereas the viscosity-dependent mechanisms associated with KGM are attenuated. The cumulative extent of lipolysis, as reported by the area under the curve (AUC), was reduced by KGM, MMT, and KGM-MMT by 6.5%, 17%, and 64%, respectively, in the fasted state (Figure 2B). The KGM-MMT hybrid combines these mechanisms, resulting in a dramatic reduction in FFA release. Under fed conditions, MMT and KGM-MMT hybrids reduced FFA release by 55% and 12%, respectively, while KGM showed no effect. It is also worth considering a potential functional interplay between KGM hydration and MMT adsorptive activity. The increased surface area of spray-dried hybrid particles may accelerate KGM hydration and network formation within the intestinal lumen, generating a viscous polysaccharide network that could partially restrict MMT platelet surfaces from accessing dietary lipids and bile acids [44]. However, under the fasted conditions evaluated in this study, KGM-MMT still outperformed MMT alone in suppressing FFA release, suggesting that this viscosity-driven hindrance does not negate the net adsorptive benefit under low-emulsifier conditions. Under fed conditions, where KGM-MMT performed less effectively than MMT alone, the reduced MMT content of the hybrid formulation combined with KGM-mediated interfacial viscosity effects may together contribute to the attenuated lipid-restrictive response. Future studies using confocal imaging of hybrid particles under simulated intestinal conditions can help clarify this mechanism. The dominance of MMT under fed conditions reflects its enhanced adsorption of lipolytic products in emulsifier-rich environments, while the hybrid’s reduced efficacy compared to the fasted state may result from its lower MMT content.

### 3.3. Restoration of the Microbiota by KGM in HFD-Fed Mice

**Figure 3:**
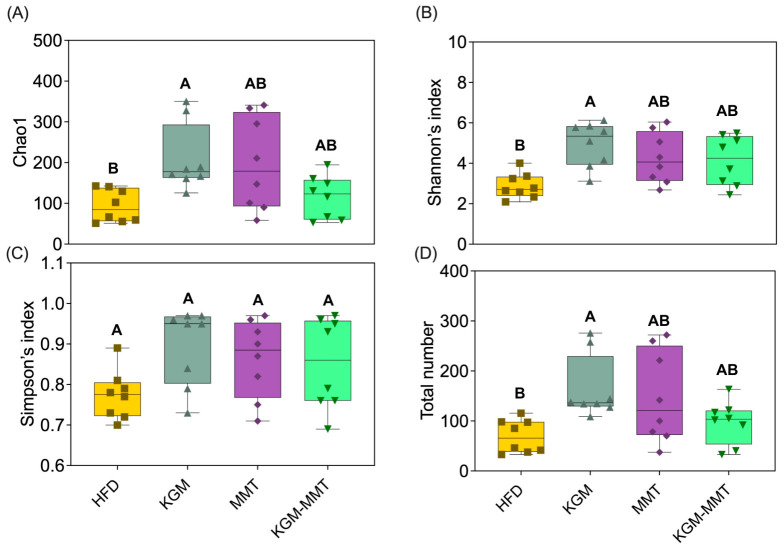
16S rRNA sequencing results highlighting microbial diversity and taxonomic composition across treatment groups. Alpha diversity indices, being (**A**) Chao1, (**B**) Shannon, (**C**) Simpson indices and (**D**) total number of operational taxonomic units, are presented as box and violin plots displaying minimum to maximum values (*n* = 8 per group), demonstrating the significant enrichment of microbial richness with KGM inclusion in the HFD. Statistical differences were determined using the Kruskal–Wallis test with Dunn’s multiple comparisons post hoc. Distinct letter notation (A, B) denotes statistically different groups, where groups sharing the same letter are not significantly different.

Treatment-dependent changes in gut microbiota composition and diversity were observed following the inclusion of KGM-MMT hybrids and their precursors in the diet of HFD-fed mice over a 42-day treatment period. Dietary supplementation with KGM significantly increased alpha diversity indices, including Chao1 richness, Shannon’s index, and total observed species counts (Figure 3A–D). KGM treatment improved these metrics by approximately 1.2- to 2.4-fold relative to the HFD group, with statistically significant differences observed for Shannon’s index (*p* = 0.0054), Chao1 richness (*p* = 0.0143), and total observed species (*p* = 0.0069). This enhancement in alpha diversity is crucial, as reduced gut microbial diversity is a recognized hallmark of gut dysbiosis and is strongly associated with metabolic disorders such as obesity, diabetes, and systemic inflammation [45]. Lower alpha diversity, particularly reductions in Chao1 and Simpson indices, has been directly linked to poorer metabolic health profiles in obese individuals and children, indicating a compromised gut ecosystem that predisposes to metabolic diseases [46]. Restoration of microbial diversity through dietary fibers such as KGM has been associated with improved metabolic resilience in obese hosts [27]. KGM modulates gut microbiota through several mechanisms. It is a high-molecular-weight, water-soluble polysaccharide with β-1,4-linked glucose and mannose and β-1,3 branches [47]. This structure resists host digestion and allows fermentation in the colon. KGM selectively enriches microbes that express mannan-degrading enzymes such as GH26 and GH134. This supports taxa within *Lactobacillaceae* and *Ruminococcaceae*, giving them a competitive advantage [48]. Fermentation produces short-chain fatty acids which lower luminal pH and suppress acid = sensitive taxa such as *Streptococcaceae* [49]. KGM also increases viscosity, slows intestinal transit, and prolongs epithelial exposure to fermentation products [32]. This promotes microbial cross feeding. Together, these mechanisms explain the shifts in microbial community structure seen in KGM-treated animals compared to HFD controls.

The increased microbial diversity supports greater ecosystem stability and resistance to pathogenic colonization, mitigating adverse outcomes such as increased intestinal permeability and systemic endotoxemia, which are triggered by Western-style, high-fat diets [50,51]. Elevated levels of lipopolysaccharides (LPS) and decreased microbiota diversity contribute additively to systemic inflammation and metabolic dysfunction [52]. These diets induce a fat-rich intestinal environment that increases LPS endotoxins, which along with other pathogenic overgrowth, reduces microbial richness and compromises intestinal epithelial integrity that ultimately triggers a systemic inflammatory response [53]. In contrast, neither MMT nor KGM-MMT hybrid treatments significantly altered Shannon’s index, Chao1 richness, Simpson’s index, or total observed species compared to the HFD group. Although slight increases were noted (~1.1- to 2.1-fold), these did not reach statistical significance (*p* > 0.16 for all comparisons). Similarly, Simpson’s index, which reflects species evenness, remained unaffected across treatments (*p* > 0.08). These data indicate that the observed diversity effects are primarily attributable to KGM’s fermentability, while MMT exerts minimal independent effects on alpha diversity.

Although spray-dried MMT and KGM-MMT hybrids increased alpha diversity indices (1.1- to 2.1-fold), these changes were not statistically significant (Figure 3A). This aligns with a previous study showing no significant change in the Shannon’s index after five weeks of daily MMT treatment (1 g/kg BW) in HFD-induced obese mice [15]. As emerging evidence suggests that restoring microbial diversity is protective against metabolic complications in obesity [46], the observed KGM-mediated improvements in alpha diversity may confer metabolic benefits.

### 3.4. Modulation of the Mice Gut Microbiota by KGM-Based Treatments

**Figure 4:**
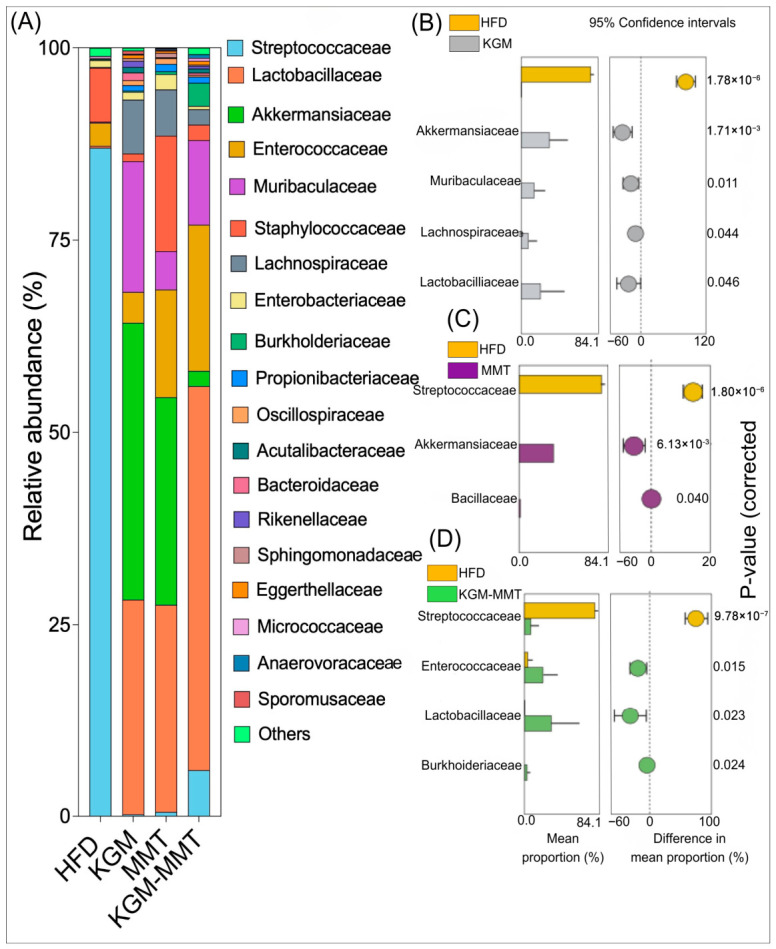
(**A**) Relative abundance of gut microbiota at the family level after the 42-day treatment period across all groups. Differential abundance analysis of family-level taxa comparing the HFD group against (**B**) KGM, (**C**) MMT, and (**D**) KGM-MMT treatments (*n* = 8 per group). Statistical significance was assessed using Welch’s *t*-test, with correction for multiple comparisons. Mean relative abundance differences between groups and their associated *p*-values are shown.

The microbiota composition analysis further highlights the deleterious effects of a HFD, which enriched harmful pathobionts such as *Streptococcaceae* (Figure 4A). In obese mice, an overgrowth of *Streptococcaceae* was linked to a 2.5-fold increase in fasting insulin levels, a 3-fold increase in serum total cholesterol, and a 3.5-fold increase in liver IL-6 mRNA expression after 8 weeks on a HFD [54]. In the current study, the dietary inclusion of KGM reduced the mean relative frequency of *Streptococcaceae* by 8.4-log_2_-fold when compared to the HFD control (*p* < 0.001) (Figure 4B). Additionally, KGM supplementation was associated with increased relative abundance of taxa previously linked to metabolic benefits, such as *Lactobacillaceae* and *Akkermansiaceae* by 5.7-log_2_-fold (*p* = 0.046) and 18-log_2_-fold (*p* = 0.0017), respectively. These families include species that have been reported to influence gut barrier integrity and host inflammatory signaling [55,56]. Another extensively researched species from the *Akkermansiaceae* family, *Akkermansia muciniphila*, can activate the nuclear factor kappa-light-chain-enhancer of the activated B cells (NF-κB) pathway in intestinal epithelial cells by releasing metabolites that induce pro-inflammatory cytokine expression. Simultaneously, it upregulates mucin 2 and tumor necrosis factor alpha-induced protein 3 (TNFAIP3), strengthening the intestinal barrier and reducing inflammation [57].

Interestingly, MMT inclusion in the mice diet also induced similar changes to *Streptococcaceae* (*p* < 0.001) and *Akkermansiaceae* (*p* = 0.0061) to those observed with KGM supplementation (Figure 4C). This suggests that MMT may indirectly promote beneficial bacterial growth. One possible mechanism is through adsorption of lipolytic products, thereby lowering luminal lipid levels, which are often linked to dysbiosis [15].

In addition to the positive microbial shifts in *Lactobacillaceae* (*p* = 0.023) and *Streptococcaceae* (*p* < 0.001)*,* KGM-MMT enhanced the relative mean frequency of *Enterococcaceae* by 2.4-log_2_-fold (*p* = 0.015) (Figure 4D). This family includes species such as *Enterococcus cecorum*, which has been linked to improved glucose and lipid metabolism in HFD-fed mice [58]. It is important to recognize that the observed microbial shifts may not be solely due to dietary inclusions. Microbial composition is dynamic and can fluctuate over time, and without longitudinal monitoring, the stability and persistence of these changes remain unclear [59]. Moreover, while certain family-level taxa are known to include species with metabolic benefits, these families may also harbor species with potentially harmful effects. Consequently, functional interpretations should not be regarded as definitive. Future studies should therefore utilize higher-resolution microbial approaches, such as metagenomic sequencing, in order to better postulate the functional consequences of these gut microbiota shifts [60].

### 3.5. HFD-Induced Microbial Composition Is Altered by KGM and MMT Treatmens

**Figure 5:**
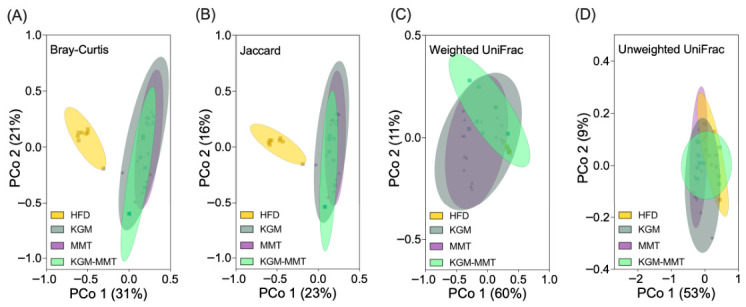
Beta-diversity PCoA plots illustrate the variation in microbial community composition between HFD enriched with KGM, MMT, or KGM-MMT (*n* = 8 per group). These plots are based on four metrics: (**A**) Bray–Curtis (abundance-based dissimilarity), (**B**) Jaccard (presence/absence-based dissimilarity), (**C**) Weighted UniFrac (phylogenetic distance accounting for relative abundances), and (**D**) Unweighted UniFrac (phylogenetic distance based on presence/absence). PCoA plots with 95% confidence ellipses were used to visualize group clustering and variability. Statistical differences in beta-diversity between groups were assessed using PERMANOVA (permutational multivariate analysis of variance).

Beta-diversity analysis highlights the differences in microbial composition between treatment groups, providing insight into how gut-active materials affect the gut microbiota of HFD-fed mice. The HFD control group exhibited a distinct community composition relative to all intervention groups, consistent with treatment-associated shifts in microbial structure (Figure 5A,B). Bray–Curtis and Jaccard PCoA with PERMANOVA indicated significant between-group differences between HFD controls and KGM (*p* = 0.00006), MMT (*p* = 0.00006), and KGM-MMT (*p* = 0.00018) groups, supporting intervention-associated divergence under HFD conditions. All groups were maintained under HFD conditions by design to enable direct comparison of treatment-specific effects under a shared metabolic stressor. Accordingly, the present microbiome analyses quantify intervention-associated shifts within an obesogenic context rather than normalization to a lean baseline. Inclusion of a chow-fed reference in future work would be valuable for benchmarking the magnitude of divergence from a healthy state. Nevertheless, the current study design allows for the ranking of the relative microbiome-modulatory effects of KGM, MMT, and the hybrid within the HFD model. The observed changes in microbial taxa suggest that KGM and MMT supplementation may help mitigate some of the gut microbiota imbalances typically associated with HFD consumption. Previous studies have demonstrated that HFDs induce significant changes in microbial community structure. HFD exposure has been associated with altered community structure in both mice and humans, including reported shifts in broad phyla- and family-level taxa; however, phylum-level summaries (e.g., *Firmicutes*/*Bacteroidetes* ratios) are not consistently predictive across studies [61]. Other studies have reported specific enrichments of families such as *Rikenellaceae* and reductions in *Lactobacillaceae* in HFD-fed mice [62].

The differences between KGM and KGM-MMT were pronounced, with the Bray–Curtis (*p* = 0.01176) and Jaccard (*p* = 0.01602) analyses indicating significant compositional divergence between these two interventions. Phylogenetic analysis using UniFrac distances, which account for phylogenetic lineages, showed no significant differences between the HFD and treatment groups (Figure 5C,D). The lack of significant separation using UniFrac metrics suggests that observed differences were driven primarily by changes in relative abundance within shared phylogenetic lineages rather than the appearance or loss of deeply divergent taxa. For example, taxa such as *Lactobacillaceae* were enriched, and *Streptococcaceae* were depleted with KGM treatment, reflecting a microbial shift within a narrow phylogenetic range. These results contrast other findings that indicate HFD can significantly alter phylum-level changes; however, these changes were most pronounced between 8 and 12 weeks of HFD-feeding in mice [63]. Collectively, these results are consistent with early-stage HFD models in which family-level abundance shifts may precede larger phylum-level restructuring reported in longer feeding durations.

### 3.6. Microbial Enzymatic Diversity Enhanced by KGM

**Figure 6:**
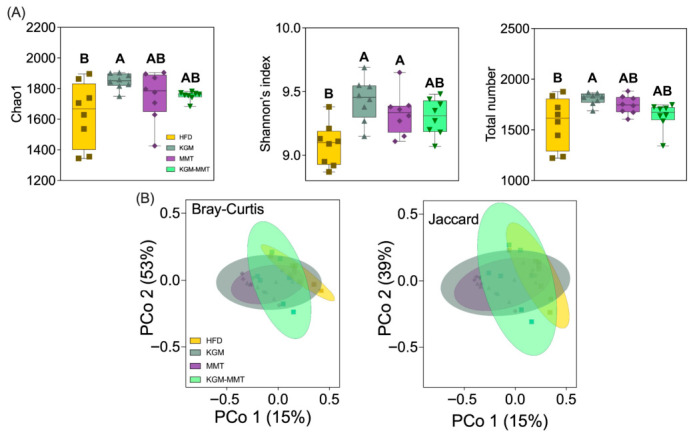
Predicted microbial enzymatic diversity and functional composition across dietary groups. (**A**) Alpha diversity indices, including Shannon’s index and total observed enzyme counts, are presented as box and violin plots displaying min to max values (*n* = 8 per group), demonstrating significant treatment-induced changes in microbial functional diversity. (**B**) Beta-diversity analysis based on Bray–Curtis and Jaccard indices reveals distinct clustering of microbial enzymatic compositions among groups. Statistical differences were determined using the Kruskal–Wallis test with Dunn’s multiple comparisons post hoc. Distinct letter notation (A, B) denotes statistically different groups, where groups sharing the same letter are not significantly different.

Functional predictions were generated using PICRUSt2 from 16S rRNA profiles to infer relative shifts in enzyme and pathway potential. These predictions serve only to infer possible correlations and are not a substitute for shotgun metagenomics or targeted metabolomics. Nonetheless, PICRUSt2 from 16S rRNA data reported a Spearman correlation in the range of 0.79 to 0.88 when compared to KO relative abundances obtained from matched shotgun metagenomes and are, therefore, strong predictors of functionality. KGM supplementation was associated with a 3.9–15% increase in predicted enzyme alpha diversity relative to HFD controls (Figure 6A). This is consistent with earlier findings in this study that highlight KGM’s role as a fermentable substrate that promotes gut microbial growth, enhancing microbial enzymatic expression in the process. Similarly, MMT supplementation demonstrated a distinct but less pronounced impact, promoting Shannon’s index by 2.7% (*p* < 0.05) (Figure 6A). The modest functional shift observed with MMT may reflect indirect ecological effects of altered luminal lipid and bile acid availability, consistent with the adsorption-driven mechanism proposed for smectite clays. To our knowledge, limited work has applied 16S-based functional prediction to isolate the enzymatic impacts of KGM or purified MMT in HFD models, emphasizing the exploratory nature of the present functional analysis. The hybrid showed a modest increase in predicted enzyme diversity (2.4%), which did not reach statistical significance (*p* = 0.0906), indicating that any functional convergence under the hybrid condition is comparatively small within this study’s duration and sample size. This result may reflect a limited study duration, as additive effects between fermentable polysaccharides and MMT could take longer to influence the current HFD-induced mice model of obesity.

Beta-diversity metrics, and Bray–Curtis and Jaccard indices, revealed distinct enzyme compositions among the dietary groups (Figure 6B). Bray–Curtis and Jaccard analyses of predicted enzyme profiles indicated significant separation of KGM and MMT from HFD controls, consistent with the observed intervention-associated compositional shifts in 16S profiles. No significant separation was observed between KGM-MMT and HFD, suggesting functional overlap and/or limited detectable effect size under the hybrid condition in this dataset.

### 3.7. KGM and MMT Enhance Enzymes and Metabolic Pathways Regulating Intestinal Barrier and Bile Acid Function

**Figure 7:**
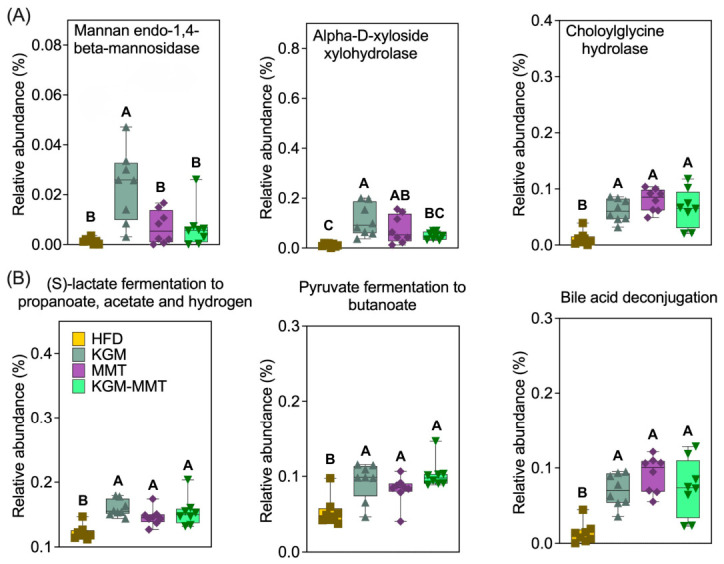
(**A**) Box and violin (minimum to maximum values) plots highlight the relative abundances of key enzymes inferred using the EC database, showing significant increases in mannan endo-1,4-beta-mannosidase, alpha-D-xyloside xylohydrolase with KGM administration, and choloylglycine hydrolase with all treatments (*n* = 8 per group). (**B**) Metabolic pathways clustered using the MetaCyc database reveal enhancements in SCFA-related fermentation pathways, including (S)-lactate fermentation to propanoate, acetate, and hydrogen, pyruvate fermentation to butanoate, and bile acid deconjugation (*n* = 8 per group). Statistical differences were determined using the Kruskal–Wallis test with Dunn’s multiple comparisons post hoc. Distinct letter notation (A, B, C) denotes statistically different groups, where groups sharing the same letter are not significantly different.

Differential analysis of predicted enzymes and pathways identified several treatment-associated shifts relevant to polysaccharide utilization, SCFA-related fermentation, and bile acid metabolism (Figure 7). As hypothesized, KGM supplementation resulted in a 17-fold increase in the abundance of mannan endo-1,4-beta-mannosidase (Figure 7A). This enzyme hydrolyzes beta-1,4 glycosidic bonds in KGM, breaking it into smaller oligosaccharides that gut bacteria can metabolize [32]. KGM also elevated the relative abundance of alpha-D-xyloside xylohydrolase, which cleaves alpha-xylosidic linkages abundant in KGM, by 9.5-fold [64]. Interestingly, MMT supplementation led to a 6.3-fold increase in the same enzyme, although this did not reach statistical significance (*p* = 0.0530) and should be interpreted cautiously. The ability of MMT to influence gut microbial enzymatic activity may be attributed to its role in adsorbing microbial by-products and altering microbial populations, as previously noted in studies examining the effects of natural clays on gut microbiota [15,16,17,19].

The relative abundance of choloylglycine hydrolase increased by 6.8-fold in mice fed MMT-enriched diets. This enzyme is involved in the hydrolysis of conjugated bile acids, which catalyzes bile acid deconjugation and may reduce micelle formation efficiency, thereby potentially lowering lipid solubilization in the intestinal lumen [65]. Choloylglycine hydrolase abundance may have been influenced by MMT’s adsorptive properties, which may interfere with bile acid availability. A similar increase was observed in the KGM-MMT hybrid diet (5.6-fold), though this effect was slightly less pronounced, likely due to the reduced proportion of MMT in the hybrid treatment. KGM alone increased choloylglycine hydrolase abundance, likely by promoting bile acid-metabolizing bacteria (e.g., *Bifidobacterium* spp. and *Lactobacillus* spp.), with *Lactobacillaceae* significantly enhanced in all treatments in the current study [66].

Metabolic pathway analysis revealed notable changes in SCFA production and bile acid metabolism across treatments (Figure 7B). All dietary interventions significantly enhanced the (S)-lactate fermentation to propanoate, acetate, and hydrogen pathway, with increases ranging from 1.2- to 1.3-fold. This pathway is critical for SCFA generation, including acetate, propionate, and butyrate, and has been linked to gut barrier-supportive phenotypes via SCFA-mediated signaling, although barrier function was not directly quantified in the present study [67]. SCFAs can also reduce inflammation by activating G-protein-coupled receptors (GPR41, GPR43) and inhibiting histone deacetylases [68]. These actions help modulate immune responses and reduce chronic low-grade inflammation often seen in obesity [69]. KGM administration resulted in the greatest increase in this pathway, followed by the KGM-MMT hybrid. This is consistent with the role of KGM as a fermentable polysaccharide, promoting the microbial production of SCFAs. Similarly, pyruvate fermentation to butanoate was significantly enhanced across all treatments, with increases ranging from 1.5- to 1.9-fold. The KGM-MMT hybrid produced the greatest enhancement, potentially due to the additive effects of MMT and KGM on microbial activity. Butyrate, a key product of this pathway, is a crucial energy source for colonocytes, fulfilling 70–80% of their energy requirements through oxidation, which also helps maintain an anaerobic environment favorable for beneficial microbiota [70,71]. Moreover, butyrate administration can decrease pro-inflammatory cytokines (e.g., IL-1β, IL-6, IL-8, TNF-α), whilst simultaneously promoting anti-inflammatory cytokines (e.g., IL-10, TGF-β) [72,73,74].

Bile acid metabolism was also significantly influenced, with bile acid deconjugation enhanced by 5.1-, 5.4-, and 6.7-fold for KGM, KGM-MMT, and MMT treatments, respectively. The potent effects of MMT-based treatments may be due to their role in bile acid restriction, reducing dietary fat availability in the process [15,16,17,19,20]. KGM also contributed to increased bile acid deconjugation, likely through its fermentation by the gut microbiota, which promotes microbial enzymes capable of bile acid metabolism [66]. The less pronounced increase observed with the KGM-MMT hybrid can be due to the lower proportion of the clay in the treatment, highlighting the dose-dependent effects of MMT on bile acid modulation. Collectively, these predictions are consistent with a model in which adsorption-driven modulation of luminal bile acids and lipids dictates microbial functionality toward increased bile acid deconjugation potential; however, causality cannot be established from prediction-based data alone.

### 3.8. KGM-MMT Reduces HFD-Induced Weight Gain

**Figure 8:**
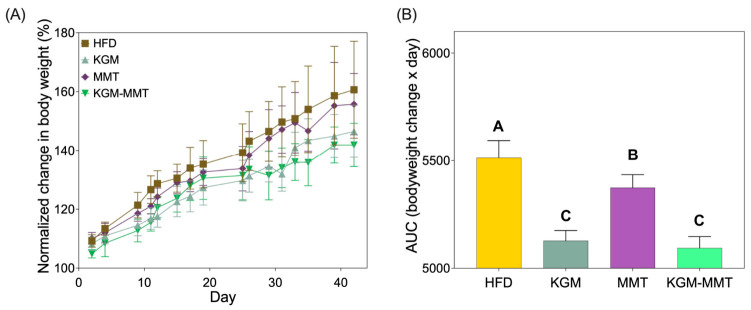
(**A**) Normalized bodyweight changes over 42 days show reduced weight gain with all treatments compared to HFD (*n* = 8 per group). (**B**) Area under the curve (AUC) analysis confirms the greatest reduction in weight gain with the KGM-MMT hybrid, followed by KGM and MMT treatments. Statistical differences were determined using one-way ANOVA with Tukey’s comparisons post hoc. Distinct letter notation (A, B, C) denotes statistically different groups, where groups sharing the same letter are not significantly different.

After 42 days of dietary intervention, all treatment groups exhibited reduced body weight gain compared to HFD-fed controls (Figure 8A). Area under the curve (AUC) analysis of body weight change confirmed this pattern, with all treatments significantly reducing cumulative body weight gain compared to HFD (*p* < 0.0001 for all comparisons). The hybrid reduced cumulative weight gain more than MMT (*p* < 0.0001) but was not statistically different from KGM (*p* = 0.6116), indicating that weight control under this dosing regimen is primarily KGM-driven, with the hybrid retaining this benefit while integrating MMT-associated mechanisms assessed elsewhere (Figure 8B). Weight gain profiles across the treatment phase remained similar across groups until approximately day 25 (Figure 8A). After this point, mice receiving KGM and KGM-MMT displayed a distinct plateau in body weight gain, whereas those in the HFD and MMT groups continued to gain weight steadily. These results align with prior findings that weight divergence between standard chow and HFD-fed rodents becomes evident after 3–4 weeks of dietary intervention [75,76]. KGM’s anti-obesity effects have been widely reported and are attributed to mechanisms that have been attributed to KGM in prior work, including delayed gastric emptying and increased viscosity, which are often associated with altered satiety signaling (e.g., GLP-1, PYY), although these hormones were not measured here [42]. Recent reports further suggest KGM can influence adipose tissue thermogenic pathways (e.g., ADRB3/UCP1 axis), although these endpoints were not assessed in this study [77].

In contrast, MMT primarily acts by adsorbing dietary lipids, bile acids, and lipolytic products in the gut, thereby reducing fat absorption and its bioavailability [15,17,18]. While this mechanism contributed to a modest reduction in weight gain, MMT was notably less effective than KGM or the KGM-MMT hybrid. Among all groups, the KGM-MMT hybrid demonstrated the greatest reduction in AUC (5094 ± 52.95), corresponding to a 7.6% decrease compared to HFD (*p* < 0.0001) and a 5.2% decrease compared to MMT (*p* < 0.0001). Although the reduction was not statistically different from KGM alone, the hybrid still delivered the lowest cumulative weight gain. This suggests a potential additive effect, likely due to the combination of KGM’s satiety-enhancing and thermogenic properties with MMT’s capacity to reduce fat absorption. This multi-mechanistic action for hybrids is supported by similar findings in other fiber-based hybrid systems. For instance, bacterial cellulose–KGM and KGM–dihydromyricetin complexes have demonstrated enhanced metabolic effects, including greater reductions in body weight, insulin resistance, and oxidative stress, than either component alone [7,78]. A limitation is the absence of a chow-fed group, which prevents comparing normalization of weight gain to a lean baseline. However, because all interventions were compared under identical HFD conditions, the present data support relative efficacy of KGM, MMT, and the hybrid in a model of obesity.

### 3.9. KGM-MMT Reduces Inflammation and Blood Glucose Whilst Improving Body Composition

**Figure 9:**
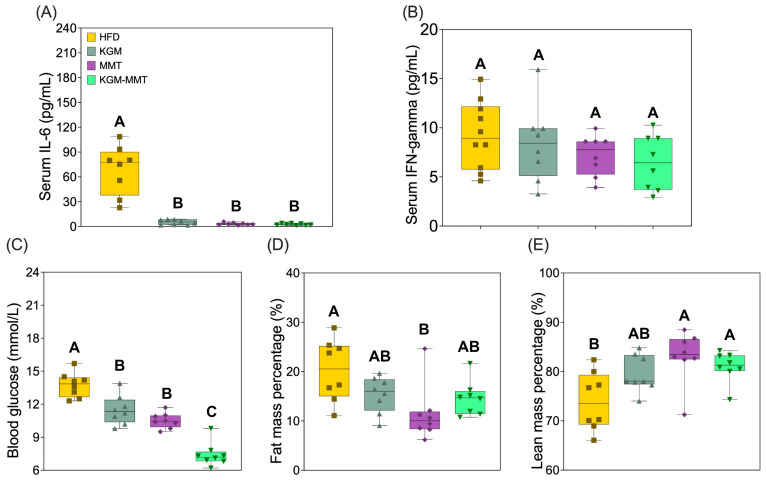
Effects of dietary treatments on systemic inflammation, blood glucose, and body composition in HFD-fed mice after 42 days. (**A**) Serum IL-6 concentrations were significantly reduced by 95% with MMT and by 97% with KGM-MMT. KGM reduced IL-6 by 93% but was not significant (*p* = 0.0555). (**B**) Serum IFN-γ levels were not significantly different across groups. (**C**) Blood glucose levels decreased by 16% (KGM), 24% (MMT, *p* = 0.0271), and 46% (KGM-MMT, *p* < 0.0001). (**D**) Fat mass percentage was significantly reduced by 44% with MMT (*p* = 0.0076), while the 29% reduction in the KGM-MMT group was not significant. (**E**) Lean mass increased by 12% with MMT (*p* = 0.0032) and by 10% with KGM-MMT (ns). Data shown as box and violin plots (min-max). *n* = 8 per group. Statistical differences were determined using the Kruskal–Wallis test with Dunn’s multiple comparisons post hoc. Distinct letter notation (A, B, C) denotes statistically different groups, where groups sharing the same letter are not significantly different.

All treatment groups showed marked reductions in serum IL-6 concentrations relative to HFD-fed controls (Figure 9A). IL-6 levels were reduced by approximately 95% in the MMT group (*p* = 0.0030) and 97% in the KGM-MMT group (*p* = 0.0002). The KGM group showed a similar directional reduction (93%), but this did not reach statistical significance (*p* = 0.0555). The large reduction in IL-6 across all groups, 93 to 97%, suggests mechanisms beyond microbiota changes alone. Several pathways are likely involved, such as reduced body weight and adipose mass lowering circulating IL-6, as adipose tissue is a major source of this cytokine in obesity [79]. MMT may also adsorb luminal LPS and other endotoxins, limiting their translocation and reducing TLR4-driven IL-6 production [15].

Predicted increases in SCFAs may contribute through GPR41 and GPR43 signaling and inhibition of histone deacetylases, which suppress NF-κB-driven IL-6 expression [80]. KGM showed a greater, but non-significant, reduction in IL-6 than MMT when normalized to body weight. This is consistent with satiety effects that reduce caloric intake and lipid burden [42]. Overall, the IL-6 reduction likely reflects both microbiota-dependent and independent mechanisms. These reductions are consistent with previous studies showing that KGM and MMT downregulate inflammatory signaling in HFD-induced obesity [7,15,16]. KGM, particularly in fiber hybrid forms, has been shown to lower hepatic IL-6 and TNF-α by 39% and 53%, respectively [7], while MMT reduces hepatic IL-6, TNF-α, and COX-2 expression, restoring inflammatory markers toward healthy levels [15,16]. Serum IFN-γ levels, however, did not differ significantly across groups in the current study (Figure 9B). While MMT and KGM-MMT demonstrated reductions (~27–29% below HFD), these changes were not statistically significant (*p* > 0.05 for all comparisons). This suggests a selective anti-inflammatory effect, with IL-6 more sensitive to dietary modulation than IFN-γ in this model. Future studies should therefore assess a broader inflammatory profile, including cytokines such as TNF-α, IL-10, and MCP-1, to better characterize systemic immune responses and their relationship with the current treatments. Intestinal barrier function was not directly measured in this study. However, indirect evidence in this study suggests barrier support. This includes predicted increases in SCFA-related pathways, enrichment of *Lactobacillaceae* and *Akkermansiaceae*, and suppression of pathobionts such as *Streptococcaceae*. These changes are linked to improved tight junction integrity and reduced permeability [81,82]. Nonetheless, direct assessment is still required. Future studies should measure barrier function using transepithelial electrical resistance (TEER), claudin and occludin expression, and fluorescein isothiocyanate dextran permeability assays (FITC dextran).

Blood glucose levels were significantly reduced in the MMT group by 24% (*p* = 0.0271) and in the KGM-MMT group by 46% (*p* < 0.0001) compared to HFD-fed controls (Figure 9C). The KGM group resulted in a 16% reduction that did not reach statistical significance (*p* = 0.3831). Moreover, glucose levels in the KGM-MMT group were significantly lower than those in the KGM group (*p* = 0.0136), consistent with additive integration of KGM- and MMT-associated mechanisms within the hybrid, rather than synergy. These effects align with established mechanisms, whereby KGM delays gastric emptying and enhances insulin signaling via upregulation of glycolytic enzymes and glycogen synthesis [8,83,84,85], while MMT reduces nutrient absorption by binding dietary lipids and bile acids [15,16]. While MMT significantly reduced fat mass (*p* = 0.0076), the hybrid showed a non-significant directional decrease (29%; *p* = 0.5908, Figure 9D,E). Thus, under the present conditions, body composition effects appear predominantly MMT-driven, with the hybrid not demonstrating an additional body composition advantage beyond MMT. Collectively, these results suggest that the KGM-MMT hybrid combines complementary mechanisms—KGM’s effects on satiety and glycemic control, and MMT’s lipid-binding and adiposity-limiting functions—to produce broad metabolic benefits.

### 3.10. KGM-MMT Hybrids Reduce Food Intake and Meal Size

**Figure 10:**
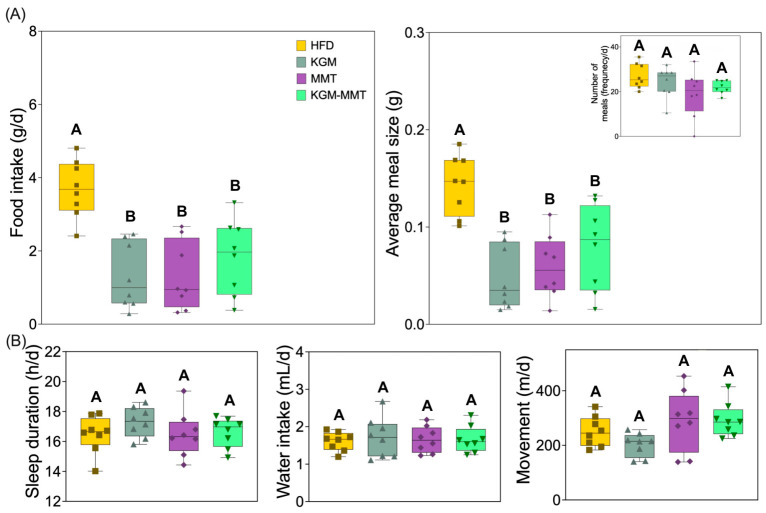
(**A**) Box and violin (min-max) plots show that all treatments reduced food intake by 51–65% and meal size by 45–66%, without altering meal frequency (inset), consistent with reductions in total intake and meal size without changes in meal frequency (*n* = 8 per group). (**B**) Sleep duration, water intake, and movement were unaffected, supporting the hypothesis that changes in feeding behavior are not stress-induced but due to physiological effects of the interventions. Statistical differences were determined using the Kruskal–Wallis test with Dunn’s multiple comparisons post hoc. Distinct letter notation (A, B) denotes statistically different groups, where groups sharing the same letter are not significantly different.

During the final week of the 42-day treatment period, mice were housed in metabolic cages to monitor feeding behavior and metabolic parameters. All treatment groups demonstrated reductions in total daily food intake compared to HFD-fed controls (Figure 10A). Mean daily intake was reduced by 65% with KGM (*p* = 0.0029), 63% with MMT (*p* = 0.0047), and 51% with KGM-MMT (*p* = 0.0681). Although the KGM-MMT group consumed slightly more than the individual components, the difference was not statistically significant.

Average meal size followed a similar pattern. Reductions of 66% (KGM) (*p* = 0.0012) and 58% (MMT) (*p* = 0.0087) were observed, while the KGM-MMT group showed a 45% decrease that was not statistically significant (*p* = 0.1409). Meal frequency was unchanged, indicating that reduced intake was driven primarily by smaller meals rather than fewer feeding episodes; however, direct satiety physiology (e.g., GLP-1/PYY or gastric emptying) was not measured. Previous studies have shown that KGM promotes satiety via increased gut viscosity, delayed gastric emptying, and elevated levels of satiety-related hormones such as Glucagon-like peptide-1 (GLP-1) and Peptide YY (PYY) [42,86,87]. The mechanisms underlying reduced intake with MMT remain unclear in this study. One plausible contribution is altered luminal lipid/bile acid availability and microbiome composition, which may secondarily influence appetite-related signaling; however, these mechanistic links remain speculative without hormone measurements or gastric emptying assays. Although MMT’s impact on gastric viscosity remains unexplored, it has been demonstrated to modulate the gut microbiota, increasing taxa such as *Akkermansia* that influence tryptophan and arginine metabolism, both linked to appetite regulation [88]. These effects could theoretically enhance satiety signaling, although this was not directly measured in the current study. Importantly, no significant differences were observed in behavioral stress indicators including sleep duration, water intake, or movement activity (Figure 10B, all *p* > 0.05). This suggests that reduced food and meal size were unlikely due to stress or altered activity and more likely reflect treatment-induced physiological effects. It must be acknowledged that the KGM-MMT hybrid did not significantly outperform KGM or MMT alone in reducing food intake or meal size. Moreover, while reduced intake and meal size support a satiety-enhancing role, the mechanistic basis remains unknown. Future studies should, therefore, directly investigate the underlying mechanisms through gastric emptying assays, as well as systemic hormonal markers (e.g., GLP-1, PYY).

## 4. Conclusions

This study shows that spray-dried KGM–MMT hybrid microparticles can be formulated as a stable oral dosage form. When incorporated into a high-fat diet, they are associated with improvements in selected metabolic outcomes in mice. The hybrid combines a fermentable polysaccharide, in KGM, with the adsorptive properties of MMT, integrating gut fermentability with adsorption-driven modulation of luminal lipids and bile acids. The hybrid reduced cumulative weight gain versus HFD controls and produced significant reductions in blood glucose and serum IL-6; directional reductions in food intake and meal size were observed but did not reach statistical significance. Collectively, these findings support additive consolidation of KGM- and MMT-associated phenotypes within a single dosage form, rather than synergy or consistent superiority over each standalone component. From a biomaterials perspective, spray drying offers a scalable route to micro-engineer fiber–clay composites with tuneable morphology and dispersion that may be advantageous for gastrointestinal delivery. However, mechanistic attribution remains limited because satiety physiology (e.g., GLP-1/PYY, gastric emptying), barrier integrity, and metabolite outputs were not directly measured, and microbiome functional insights were derived from 16S-based prediction. Key limitations include the absence of a chow-fed baseline, use of male mice only, reduced post-QC microbiome sample size (*n* = 8/group), and reliance on predictive functional profiling. Future studies should evaluate dose–response, longer durations with longitudinal sampling, the safety and tolerability of repeated clay exposure, and validation of predicted functional shifts using targeted bile acid/SCFA quantification and/or metagenomics prior to the consideration of translational applicability. Additionally, fecal fat content was not quantified in the present study, which limits direct mechanistic attribution of MMT-associated body weight and adiposity effects to luminal fat adsorption and excretion. The measurement of fecal lipid output in future work would directly support the proposed fat-binding mechanism of MMT. Future material characterization studies should additionally include comparisons with unprocessed KGM and MMT precursors alongside their spray-dried equivalents, to assess any benefits conferred by spray drying KGM-MMT.

## Data Availability

All data analyzed during this study are available from the corresponding author upon request.

## Abbreviations

The following abbreviations are used in this manuscript:

ADRB3	Adrenergic receptor beta 3
ARRIVE	Animal Research: Reporting of In Vivo Experiments
AUC	Area under the curve
BW	Body weight
COX-2	Cyclooxygenase-2
EC	Enzyme Commission
ELISA	Enzyme-linked immunosorbent assay
FFA	Free fatty acid
FaSSIF	Fasted state simulated intestinal fluid
FeSSIF	Fed state simulated intestinal fluid
GLP-1	Glucagon-like peptide-1
GPR	G-protein-coupled receptor
HFD	High-fat diet
IFN-γ	Interferon-gamma
IL	Interleukin
KGM	Konjac glucomannan
MMT	Montmorillonite
KO	KEGG ortholog
LPS	Lipopolysaccharide
MCP-1	Monocyte chemoattractant protein-1
MCT	Medium-chain triglyceride
MetaCyc	Metabolic Pathway Database
NF-κB	Nuclear factor kappa-light-chain-enhancer of activated B cells
NHMRC	National Health and Medical Research Council
OTU	Operational taxonomic unit
PCoA	Principal coordinates analysis
PERMANOVA	Permutational multivariate analysis of variance
PICRUSt	Phylogenetic Investigation of Communities by Reconstruction of Unobserved States
PYY	Peptide YY
SCFA	Short-chain fatty acid
SEM	Scanning electron microscopy
TGF-β	Transforming growth factor beta
TNF-α	Tumor necrosis factor alpha
TNFAIP3	Tumor necrosis factor alpha-induced protein 3
UCP1	Uncoupling protein 1
